# Congenital nasal pyriform aperture stenosis: a rare cause of neonatal nasal airway obstruction

**DOI:** 10.1259/bjrcr.20150006

**Published:** 2015-04-20

**Authors:** A Rao, S M Godehal, A R Patil, G Mallarajapatna, S Nandikoor, M Hariharan

**Affiliations:** Department of Radiology, Apollo Hospitals, Bangalore, Karnataka, India

## Abstract

Congenital nasal pyriform aperture stenosis (CNPAS) is a rare cause of nasal airway obstruction that clinically mimics choanal atresia in a neonate. The differentiation between the two is very important as the management of the two conditions is different. Timely recognition is important to prevent fatal outcome. CNPAS may present as an isolated condition or with associated craniofacial anomalies. Despite typical findings of CNPAS being present on cross-sectional imaging, this condition is commonly overlooked, probably because of a lack of familiarity with the normal morphological features of the nasal cavity in infants and also owing to a lack of awareness of this rare entity. Here we report a case of CNPAS with pre- and post-surgical CT images and the complication that occurred owing to nasal stenting.

Congenital nasal pyriform aperture stenosis (CNPAS), first published in the radiology literature in 1988 and described clinically in 1989,^1^ is one of the rare causes of neonatal airway obstruction, occurring at a frequency of one-fifth to one-third that of choanal atresia.^2^

Congenital airway obstruction is a problem that affects up to 1 in 5000 infants.^1,3^ A majority of these obstructions result from choanal atresia,^1^ which affects 1 in 8000 live births. The prevalence of CNPAS is unknown.^4^ In choanal atresia, the posterior nasal cavity is obstructed by a bony or membranous plate. However, in CNPAS the anterior nasal cavity is narrowed but patent. Immediate recognition and appropriate therapy are important for this potential life-threatening condition.^5^ Although the diagnosis of CNPAS is suggested by physical examination, the final diagnosis is made by a CT scan of the nasal cavity.^1^

## CASE REPORT

**Figure 1.**
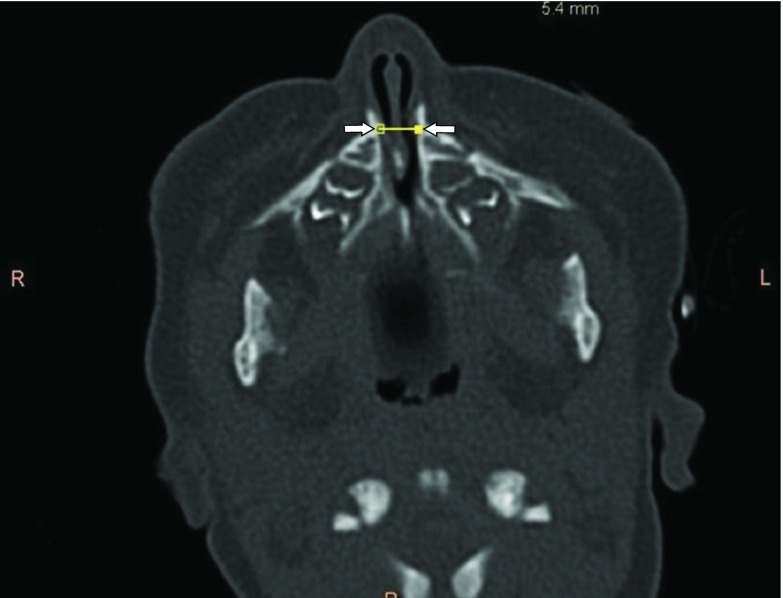
CT image (pre-stenting): axial view of the narrowed pyriform aperture measuring 5.4 mm. Note the medially angled maxillary spines (white arrows).

**Figure 2.**
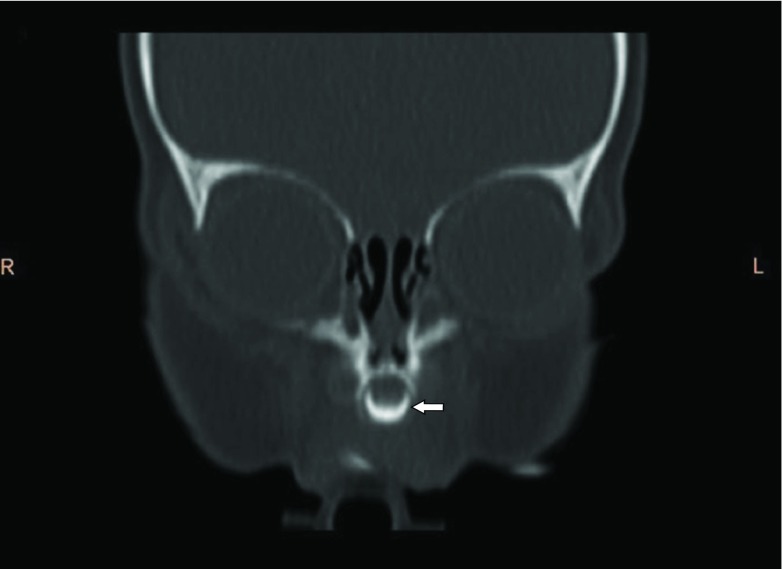
CT image (pre-stenting): coronal view showing central mega-incisor (white arrow).

**Figure 3.**
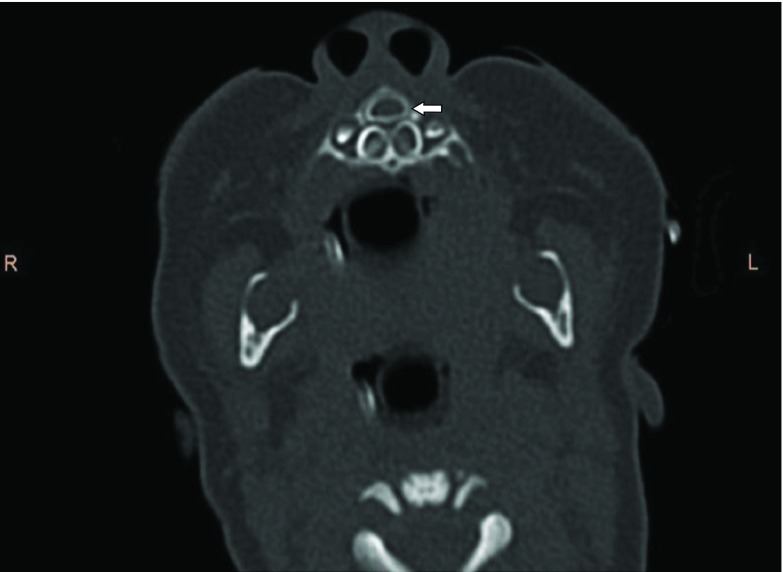
CT image (pre-stenting): axial view of the central mega-incisor (white arrow).

A 4-month-old female infant presented to our ear, nose and throat department with nasal stenting for CNPAS, which was performed elsewhere, with the stents in place. On clinical examination, the stents were seen in the bilateral nares, with partial absence of the nasal septum. Previous CT images were obtained from the parents and reviewed. CT images showed a narrowed pyriform aperture measuring 5.4 mm (Figure 1). Mild medial angulation of the nasal processes of the maxilla was noted, and a single central mega-incisor was also noted (Figures 2 and 3). CT scan of the brain showed no intracranial abnormalities.

**Figure 4.**
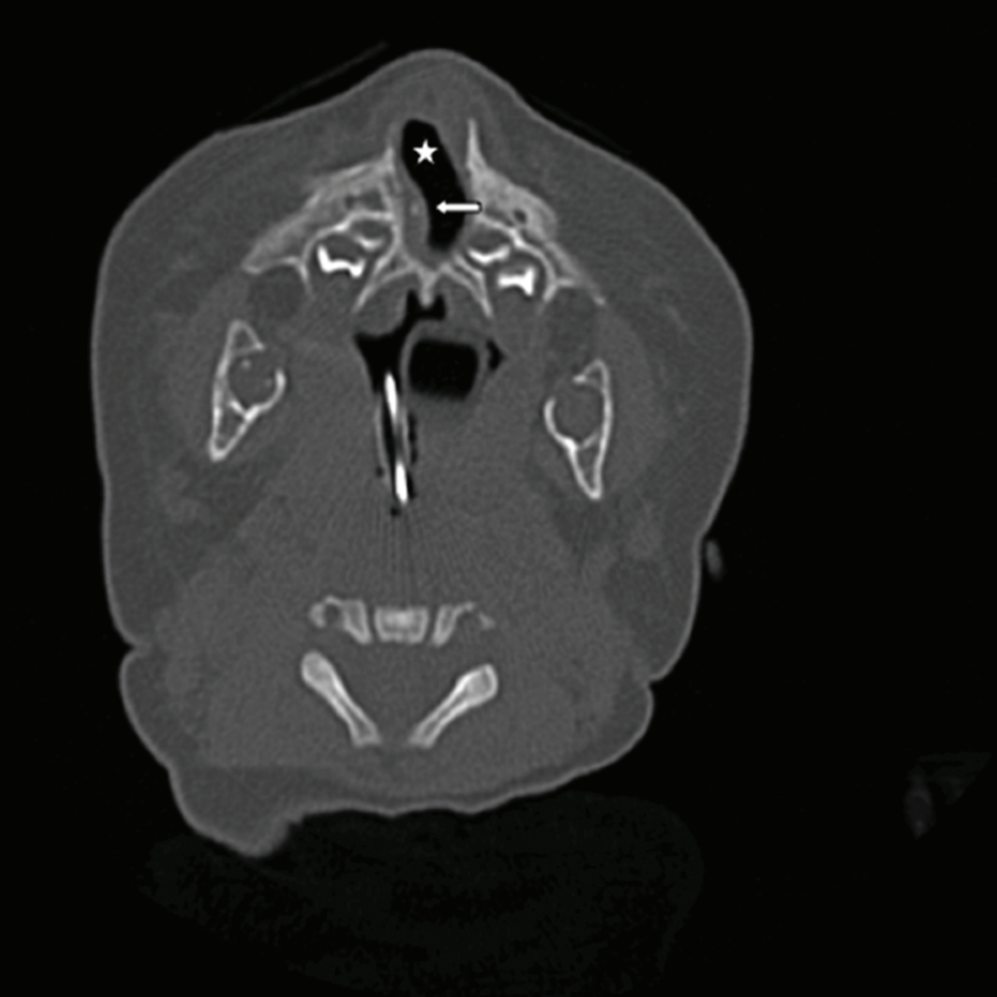
CT image (post-stenting): axial view of the widened pyriform aperture (star) measuring 10.3 mm. Nasal septum was partly eroded (white arrow).

**Figure 5.**
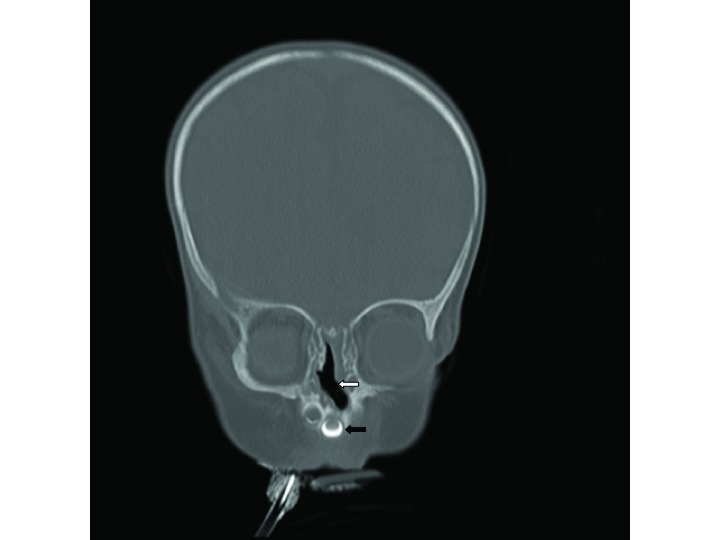
CT image (post-stenting): coronal view of the eroded septum (white arrow) and the central mega-incisor (black arrow).

**Figure 6.**
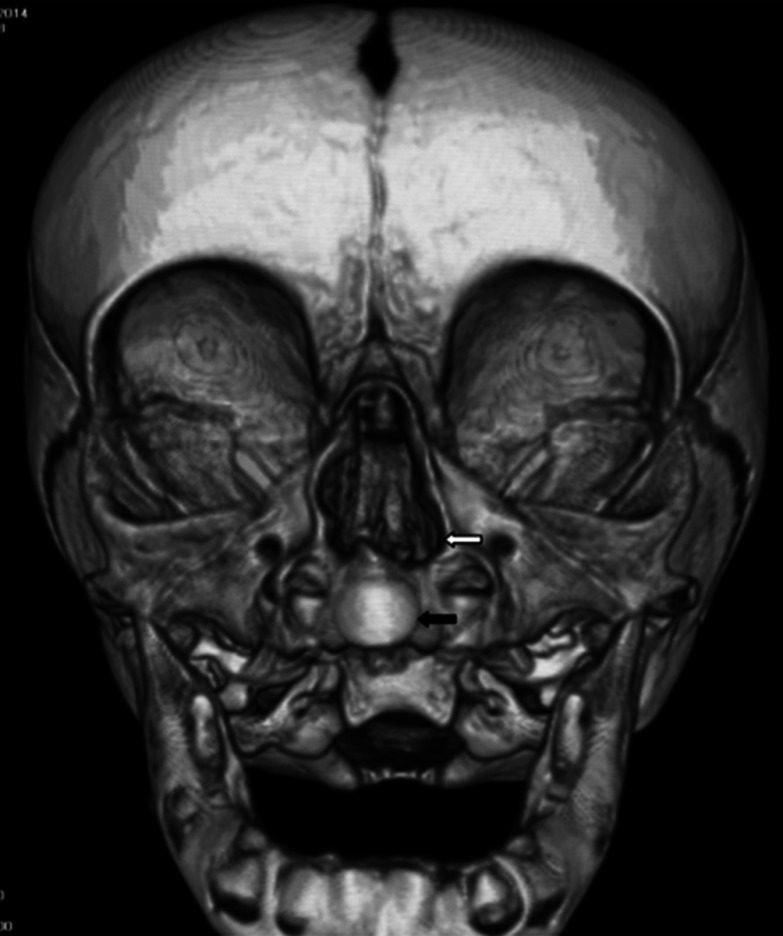
Reconstructed CT image of the widened cavity, post stenting (white arrow) with central mega-incisor (black arrow).

As the surgeons wanted to know the patency of the airway after stent removal and plan for further surgical management, the child was subjected to a CT scan immediately after removal of the stents. These CT images showed a pyriform aperture measurement of 10.3 mm (considered satisfactory; Figure 4). A central mega-incisor was noted. The nasal septum was almost completely eroded, which was probably attributed to compression erosion by the stents (Figures 5 and 6). On posterior rhinoscopy, no significant stenosis was seen at the posterior choanae. After removal of the stents, the infant was observed for cyanosis, but no complications were noted. Hence she was discharged and was asked to come back for follow-up.

## Discussion

CNPAS can clinically mimic choanal atresia, with infants presenting with respiratory distress, either at birth or within the first few months of life. The neonate can present at birth with cyanosis, breathing difficulty (particularly when feeding) and nasal cavity narrowing to the extent that passage of a nasogastric tube is impossible. Sometimes patients present with repeated episodes of cyanosis and respiratory distress until the diagnosis is made.^1^ The inability to pass a 5-Fr catheter should raise suspicions of CNPAS.^5^

Other differentials of neonatal respiratory distress, more commonly meconium aspiration, hyaline membrane disease, infection and other craniofacial malformations, should first be excluded.^5^ Traumatic causes of neonatal nasal obstruction, such as subluxated septum and septal haematoma, need to be ruled out.^6^ Skull base defects (meningoencephalocele), tumoral processes (glioma, hemangioma, teratoma and rhabdomyosarcoma) and dacryocystoceles are the other differentials.^6^

**Figure 7.**
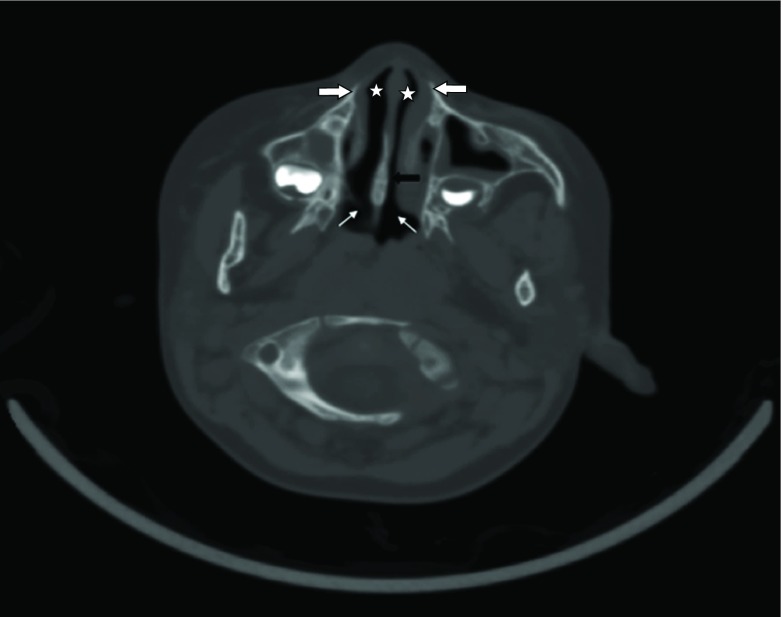
Transverse CT image obtained through the nasal cavity in a normal child. Thick white arrows indicate normal nasal processes. Note the calibre of the normal pyriform apertures (stars). Thick black arrow indicates nasal septum. Thin white arrows indicate posterior choanae.

The pyriform aperture is the narrowest part of the normal nasal airway, and small changes in its cross-sectional area can result in a significant increase in nasal airway resistance.^2^ Anatomically, the pyriform aperture is a pear-shaped bony inlet of the nose bounded laterally by the nasal processes of the maxilla, inferiorly by the junction of the horizontal processes of the maxilla and the anterior nasal spine and superiorly by the nasal bones.^5,7^ The maxillary spines mark the inferior margin of the pyriform aperture (Figure 7).^7^ The posterior choanae, or the opening between the nasal cavity and the nasopharynx, should be at least 0.34 cm in children under 2 years.^7^

The cause of this pathology is not clearly explained, but it is considered to result from an overgrowth of the nasal process of the maxilla.^5^ Two theories about the pathogenesis of CNPAS have been proposed: (1) deficiency of the primary palate, associated with a triangular hard palate, and (2) bony overgrowth of the nasal process of the maxilla, with a normal hard palate.^2^ The former theory also explains the abnormal incisors, narrow inferior portion of the nasal cavity and triangular palate. The primary palatal deficiency should also explain the abnormal incisors, narrow inferior portion of the nasal cavity and triangular palate.^1^ As a result of deficiency of the primary palate, the palatal shelves develop close to the midline, subsequently overlapping and creating a ridge along the inferior aspect of the palate in patients with CNPAS. Therefore, the degree of narrowing is greatest in the anterior aspect.^1^

Associations include craniofacial anomalies, such as holoprosencephaly, cleft palate and presence of maxillary central mega-incisors. This rare anatomical condition can be associated with choanal atresia.^5^ The old adage “face predicts the brain” holds true and hence any midline facial defects should prompt thorough evaluation of the brain.

For CT diagnosis, good, thin section (1.5 mm) images should be obtained with axial sections parallel to the anterior hard palate, as apparent narrowing may be caused by oblique imaging.^2^ The normal range of the width of pyriform sinus in the age group of 0–6 months is 8.8–17.2 mm (median width 13.5 mm). A pyriform aperture width <3 mm, or a whole pyriform aperture width <8 mm, in a term infant confirms the diagnosis of CNPAS.^2,6^ In the study by Belden et al,^1^ performed using the CT scans of six infants with CNPAS over 6 years, all six patients had a pyriform aperture width of <8 mm. This single measurement is useful in making the diagnosis of CNPAS.

The width of the pyriform aperture is taken as the total width between the medial aspects of the maxilla at the level of the inferior meatus.^1^ In addition, the area of the pyriform aperture can be obtained on coronal images by tracing the bony outline. The height of the nasal cavity is essentially normal in patients with CNPAS. Hence width measurements are sufficient for diagnosis. Thus, CNPAS can be described as an anomaly that results in narrowing of the nasal cavity that is most severe in its anterior and inferior aspects.^1^

The cause of the narrowed pyriform aperture is, most commonly, a medial deviation of the lateral wall of the pyriform aperture and, less commonly, thickening of the lateral wall.^1^

Two forms of CNPAS have been described: (a) an isolated form and (b) a form associated with other anomalies, including a midface dysostosis with central nervous system and endocrine abnormalities (pituitary defect) and central mega-incisors.^2^ Dandy–Walker malformation, hypospadias, atrial and ventricular septal defects, transethmoidal encephalocele and ambiguous genitalia have been described in association with CNPAS by Belden et al.^1^ Other associations, such as rocker bottom foot, brachycephaly, retrognathia, epicanthic folds and corpus callosal dysgenesis, have been described by Johnson and Smith.^3^ The presence of a large, single central incisor is an indication that an MRI scan of the brain should be performed to rule out holoprosencephaly. However, the large single central incisor may also be observed as an isolated anomaly in the absence of holoprosencephaly.^8^

Once identified, the initial management involves the establishment of a secure airway using a McGovern nipple^3^ or endotracheal intubation.^5^ In mild CNPAS, the approach is non-surgical, which involves the placement of silastic stents and the use of local decongestants. However, as the nasal stents are small in dimensions, they can get occluded and make daily cleaning difficult. Soft tissue injury can occur (as in our case) during cleaning and repositioning and if retained for a long period of time.

A diameter of 5 mm or more at the level of the inferior meatus on a CT scan at birth indicates chances of successful conservative management.^5^

When there is moderate-to-severe stenosis, a surgical approach is preferred. A diameter of <5 mm at the inferior meatus and patient not responding to conservative treatment require surgical treatment, which involves pyriform aperture enlargement through an endo-oral sublabial approach to reshape the stenotic area with burs. This is a good and safe method that provides adequate field exposure, preventing damage to nasolabial soft tissues without visible scarring. Good results are achieved immediately after surgery. Another method is the transnasal approach, but it is not recommended owing to the reduced dimensions of the nasal anatomical structures, which increases soft tissue injury. After surgery, the aperture is considered satisfactory if it allows the passage of a 3.5-mm endotracheal tube stent. The bony procedures should be performed anterior to the inferior turbinate to avoid nasolacrimal duct injury. When choanal atresia is associated with CNPAS, excess membrane and bony tissue should also be removed. Endoscopy is currently used for safe control of the posterior nasal fossa and to position the stents.

Nasal stents are used to reduce the recurrence of CNPAS and scar-related stenosis. In cases of isolated CNPAS, short nasal conformers are used, which are easier to clean and replace. In cases of choanal atresia also, longer, soft silastic nasal stents are mandatory to prevent obstruction of the posterior nasal area and to maintain the stability of the surgical enlargement. Stents are retained for only 6–7 days in cases of isolated CNPAS and for 3 weeks in cases associated with choanal atresia.^5^

The prognosis for CNPAS patients is excellent.

## Conclusions

The main objective of this report is to increase awareness among radiologists about this rare, potentially life-threatening condition in neonates. Key factors in identifying this condition on cross-sectional imaging should be known to confirm the diagnosis and guide the surgeons to plan further management.

## LEARNING POINTS

1. CNPAS is a rare cause of nasal airway obstruction that clinically mimics choanal atresia in a neonate and differentiation between the two is critical as the management is different.
2. Familiarity with normal morphological features of infant nasal cavity on imaging (CT scan) is helpful in distinguishing between the two.
3. Pyriform aperture width <8 mm in a term infant confirms the diagnosis of CNPAS.
4. Immediate recognition on cross-sectional imaging is crucial to guide appropriate clinical management for this potentially life-threatening condition.
5. Presence of a large, single central incisor is an indication that an MRI scan of the brain should be performed to rule out holoprosencephaly. However, the large, single central incisor may also be observed as an isolated anomaly in the absence of holoprosencephaly.
